# Obesity risk is associated with altered cerebral glucose metabolism and decreased μ-opioid and CB_1_ receptor availability

**DOI:** 10.1038/s41366-021-00996-y

**Published:** 2021-11-02

**Authors:** Tatu Kantonen, Laura Pekkarinen, Tomi Karjalainen, Marco Bucci, Kari Kalliokoski, Merja Haaparanta-Solin, Richard Aarnio, Alex M. Dickens, Annie von Eyken, Kirsi Laitinen, Noora Houttu, Anna K. Kirjavainen, Semi Helin, Jussi Hirvonen, Tapani Rönnemaa, Pirjo Nuutila, Lauri Nummenmaa

**Affiliations:** 1 Turku PET Centre, University of Turku, Turku, FI-20521 Finland; 2 Clinical Neurosciences, Turku University Hospital, Turku, FI-20521 Finland; 3 Department of Endocrinology, Turku University Hospital, Turku, FI-20521 Finland; 4 Turku PET Centre, Turku University Hospital, Turku, FI-20521 Finland; 5 Turku PET Centre, Åbo Akademi University, Turku, FI-20500 Finland; 6 MediCity Research Laboratory, University of Turku, Turku, FI-20500 Finland; 7 Turku Bioscience Centre, University of Turku and Åbo Akademi University, Turku, FI-20500 Finland; 8 Institute of Biomedicine, Research Centre for Integrative Physiology and Pharmacology, University of Turku, Turku, FI-20500 Finland; 9 Department of Radiology, University of Turku and Turku University Hospital, Turku, FI-20500 Finland; 10 Department of Medicine, University of Turku, Turku, FI-20500 Finland; 11 Department of Psychology, University of Turku, Turku, FI-20500 Finland

**Keywords:** Risk factors, Obesity, Obesity

## Abstract

**Background:**

Obesity is a pressing public health concern worldwide. Novel pharmacological means are urgently needed to combat the increase of obesity and accompanying type 2 diabetes (T2D). Although fully established obesity is associated with neuromolecular alterations and insulin resistance in the brain, potential obesity-promoting mechanisms in the central nervous system have remained elusive. In this triple-tracer positron emission tomography study, we investigated whether brain insulin signaling, μ-opioid receptors (MORs) and cannabinoid CB_1_ receptors (CB_1_Rs) are associated with risk for developing obesity.

**Methods:**

Subjects were 41 young non-obese males with variable obesity risk profiles. Obesity risk was assessed by subjects’ physical exercise habits, body mass index and familial risk factors, including parental obesity and T2D. Brain glucose uptake was quantified with [^18^F]FDG during hyperinsulinemic euglycemic clamp, MORs were quantified with [^11^C]carfentanil and CB_1_Rs with [^18^F]FMPEP-*d*_*2*_.

**Results:**

Subjects with higher obesity risk had globally increased insulin-stimulated brain glucose uptake (19 high-risk subjects versus 19 low-risk subjects), and familial obesity risk factors were associated with increased brain glucose uptake (38 subjects) but decreased availability of MORs (41 subjects) and CB_1_Rs (36 subjects).

**Conclusions:**

These results suggest that the hereditary mechanisms promoting obesity may be partly mediated via insulin, opioid and endocannabinoid messaging systems in the brain.

## Introduction

Prevalence of obesity has more than doubled from 1975 to date, and in 2016, there were over 600 million obese adults globally [1]. Obesity is a major cause of mortality and morbidity worldwide because it is accompanied with conditions such as type 2 diabetes (T2D), cardiovascular disease and neurodegeneration [2, 3]. Epidemiological studies have indicated overweight, physical inactivity, low socioeconomic status, parental obesity and parental T2D to be key risk factors for future obesity [4–9]. Energy balance regulation is a complex process controlled by both central and peripheral neurohumoral mechanisms, and brain’s reward and appetite circuits play a key role in maintenance of obesity [10]. Yet, it is not known if alterations in these systems predispose to subsequent development of obesity.

Among the peripherally produced metabolic hormones, insulin regulates pleasure-driven feeding in mesolimbic pathways, interacting with opioidergic reward systems [11]. In morbidly obese subjects, increasing the plasma insulin concentration to supraphysiological levels results in acceleration of central glucose metabolism [12]. One human positron emission tomography (PET) study with [^18^F]FDG found that middle-aged subjects with peripheral insulin resistance have blunted glucose metabolism response to insulin also in brain, especially in appetite-controlling regions such as ventral striatum [13]. These studies suggest that cerebral insulin resistance is a pathophysiological trait in developed obesity, but it is unknown whether alterations in brain’s insulin signaling could increase the risk of future weight gain in the non-obese state.

Endogenous opioids and particularly μ-opioid receptor (MOR) ligands mediate reward and are involved in the control of food intake [14]. Opioid receptor agonists stimulate and antagonists reduce food intake in rodents and humans [15]. PET studies have found global MOR downregulation in morbidly obese humans, and shown that this downregulation recovers after weight loss following bariatric surgery [16]. However, it is not known whether the initial MOR downregulation is caused by obesity, or whether it reflects a vulnerable endophenotype for excessive eating and weight gain.

In addition to opioids, endocannabinoids influence feeding through hypothalamic and cortico-limbic circuits [17]. CB_1_ receptor (CB_1_R) is the most abundant endocannabinoid receptor in the brain, modulating central effects of endogenous and exogenous cannabinoids [18]. In rats, CB_1_-agonist administration to nucleus accumbens shell increases food intake [19], whereas CB_1_R knockout mice are immune to diet-induced obesity [20] and have dampened sensitivity to food reward [21]. Furthermore, central CB_1_R density is reduced in obese rodents [22]. Lowered central CB_1_R density could thus also constitute a risk factor for developing obesity.

In this triple-tracer PET study, we investigated whether risk factors for obesity are associated with insulin-stimulated brain glucose uptake (BGU) and central MOR and CB_1_R availability in a sample of healthy, non-obese young males (*n* = 41). Obesity risk was indexed by parental obesity and T2D and participant’s physical exercise and actual BMI. The work was a part of PROSPECT project which was preregistered to Clinicaltrials.gov (Neuromolecular Risk Factors for Obesity, PROSPECT, NCT03106688). Based on prior clinical studies on obesity, we hypothesized that higher obesity risk would be associated with increased BGU during hyperinsulinemia [12]. We also hypothesized that higher obesity risk would associate with reduced MOR and CB_1_R availability [22, 23].

## Subjects and methods

The study was conducted in accordance with the Declaration of Helsinki. The Ethical Committee (EC) of the Hospital District of South-Western Finland approved the study, and all participants signed EC-approved written informed consent forms before data gathering.

### Subjects

We recruited 43 men with low or high risk for developing obesity via Internet discussion forums, traditional bulletin boards, university-hosted email lists and newspaper advertisements. Clinical screening was done by a physician (TaK or LP), and it involved medical history checkup, physical examination, 2-h oral 75 g glucose tolerance test (OGTT), urine drug-screening and blood tests. None of the subjects had detectable levels of 11-Nor-9-carboxy-Δ^9^-tetrahydrocannabinol in their blood (a marker of cannabis consumption).

Exclusion criteria were poor compliance with the study schedule, smoking or use of nicotine products, abusive use of alcohol, use of illicit drugs, any chronic disease or medication that could affect glucose metabolism or neurotransmission, neurological or psychiatric disease, eating disorder, any contraindication to magnetic resonance imaging (MRI) and prior participation in PET studies or other significant exposure to radiation. Inclusion criteria for the high-risk (HR) group were male sex, age of 20–35 years, overweight i.e., body mass index (BMI) of 25–30 kg/m^2^, leisure time physical exercise <4 h/week, maternal / paternal overweight or obesity or maternal/paternal T2D. Inclusion criteria for the low-risk (LR) group were male sex, age of 20–35 years, normal weight i.e., BMI of 18.5–24.9 kg/m^2^, leisure time physical exercise ≥4 h/week, and no maternal / paternal T2D.

Risk grouping was based on previously established risk factors for future obesity: BMI [4], leisure time physical exercise (hours/week) [8] and familial obesity risk (Family Risk i.e., current parental overweight / obesity and T2D; [7, 9] Table 1). Altogether 19 men were recruited to the HR group and 24 men to the LR group. Subjects’ body fat percentage was measured with air displacement plethysmograph (the Bod Pod system, software version 5.4.0, COSMED, Inc., Concord, CA, USA). Sample size was determined by a priori power analysis based on our prior neuroreceptor PET studies on obesity [23], which suggested that a sample size of 16 + 16 would be sufficient for establishing the predicted effects at p < 0.05 with actual power exceeding 0.95, assuming regional effect size of *r* = 0.5.

**Table 1:** The principles of familial obesity risk (Family Risk) scoring, total score ranging from 0 to 4. Gestational diabetes (one subject) was scored as type 2 diabetes.

Parent overweight or obesity	No	One parent	Both parents
	0	1	2
Parent type 2 diabetes	No	One parent	Both parents
	0	1	2

Two LR subjects were excluded after the screening because they did not respond to further contact attempts. The final sample (*n* = 41) consisted of 19 HR individuals and 22 LR individuals, who were scanned with [^11^C]carfentanil and MRI. One LR subject discontinued the [^18^F]FDG-PET study before the brain scan, because the cannulas felt unpleasant. Two LR subjects´ [^18^F]FDG scan had to be discontinued before the brain scan because of scheduling problems. A total of 19 LR and 19 HR subjects thus completed the brain [^18^F]FDG study. Due to scheduling problems and technical issues, 36 subjects (16 HR and 20 LR individuals) completed the [^18^F]FMPEP-*d*_*2*_ scan successfully. One HR subject did not arrive to the body composition analysis. Basic characteristics of the sample are summarized in Table 2.

**Table 2:** Characteristics of the final sample (*n* = 41). *p* value is for two-tailed independent samples *t* test between the two groups. 34 subjects (18 low-risk and 16 high-risk subjects) had no data points missing. The missing data are denoted and specified^a,c,d^.

	Low-risk males (*n* = 22)		High-risk males (*n* = 19)		*p* value
	mean	SD	mean	SD	
Age (years)	23.0	2.9	27.1	4.3	<0.001
BMI (kg/m^2^)	22.0	1.9	27.2	1.9	<0.001
Body fat (%)^a^	16.4	5.5	29.1	7.8	<0.001
Physical exercise (hours/week)	6.2	2.8	2.7	1.0	<0.001
Family Risk score (0–4)	0.1	0.3	1.4	0.9	<0.001
Homeostatic Model Assessment for Insulin Resistance (HOMA-IR)^b^	1.2	0.7	2.2	0.8	<0.001
Fasting plasma glucose (mmol/l)	4.9	0.5	5.5	0.4	<0.001
2-h plasma glucose in oral glucose tolerance test (mmol/l)	4.8	1.0	5.9	1.4	0.004
Injected activity of [^11^C]carfentanil (MBq)	244.5	10.7	252.6	10.7	0.02
Injected activity of [^18^F]FDG (MBq)^c^	153.7	10.3	159.4	8.9	0.08
Injected activity of [^18^F]FMPEP-*d*_*2*_ (MBq)^d^	188.2	11.0	187.6	14.8	0.88

^a^Body fat percentage for high-risk subjects is computed with *n* = 18, since one high-risk subject didn’t complete the body composition analysis.

^b^HOMA-IR indexes body insulin resistance and is quantified from fasting blood values with the equation: HOMA-IR = (fP-Glucose × fP-Insulin)/22.5.

^c^Mean and SD for the low-risk (*n* = 19) and high-risk subjects (*n* = 19) that completed the [^18^F]FDG scan successfully.

^d^Mean and SD for the low-risk (*n* = 20) and high-risk subjects (*n* = 16) that completed the [^18^F]FMPEP-*d*_*2*_ scan successfully.

### Radiochemistry

BGU was quantified with [^18^F]FDG, which was produced using FASTlab synthesis platform (GE Healthcare) according to a modified method of Hamacher et al. [24] and Lemaire et al. [25]. Radiochemical purity was >98%.

MOR availability was measured with radioligand [^11^C]carfentanil [26], which was synthesized using [^11^C]methyl triflate, where cyclotron-produced [^11^C]methane was halogenated by gas phase reaction into [^11^C]methyl iodide [27] and converted online into [^11^C]methyl triflate [28]. The [^11^C]methane was produced at the Accelerator Laboratory of the Åbo Akademi University, using the ^14^N(p,α)^11^C nuclear reaction in a N_2_-H_2_ target gas (10 % H_2_). [^11^C]methyl triflate was bubbled into a solution containing acetone (200 µl), O-desmethyl precursor (0.3–0.4 mg, 0.79–1.05 µmol) and tetrabutylammonium hydroxide (aq) (4 µl, 0.2 M) at 0 °C. The reaction mixture was diluted and loaded into a solid phase extraction cartridge (C18 Sep-Pak^®^ Light, Waters Corp., Milford, MA) and the cartridge was washed. Dilution and washing were done using 25% ethanol in sterile water solution, 10 mL each step. The [^11^C]carfentanil was extracted with ethanol from the cartridge, diluted with 0.1 M phosphate buffer solution into <10% ethanol level and finally sterile filtered (Millex GV, 0.22 µm polyvinylidene fluoride membrane, 33 mm, Merck Millipore). Analytical HPLC column (Phenomenex Luna^®^ 5 µm C8(2) 100 Å, 4.6 × 100 mm), acetonitrile (32.5%) in 50 mM H_3_PO_4_ mobile phase, 1 ml/min flow rate, 7 min run time and detectors in series for UV absorption (210 nm) and radioactivity were used for determination of identity, radiochemical purity and mass concentration. Radiochemical purity of the produced [^11^C]carfentanil batches was 98.5 ± 0.3% (mean ± SD). The injected [^11^C] Carfentanil radioactivity was 248 ± 11 MBq and molar radioactivity at time of injection 290 ± 110 MBq/nmol corresponding to an injected mass of 0.40 ± 0.23 µg.

CB_1_R availability was measured with [^18^F]FMPEP-*d*_*2*_, which was produced as described previously [29]. The radiochemical purity was >95% and the molar activity >500 GBq/μmol at the end of synthesis.

### Image acquisition

Subjects had a 12-h overnight fast before the [^18^F]FDG scan, and fasted 6–12 h before the [^11^C]carfentanil and [^18^F]FMPEP-*d*_*2*_ scans. The PET scans were done on separate days. The subjects were advised to abstain from physical exercise in the PET scan days and the day before. Detailed scan protocols and hyperinsulinemic euglycemic clamp execution are described in Supplementary Text 1. The [^18^F]FDG scans were done with GE Discovery (Discovery 690 PET/CT, GE Healthcare), and the [^11^C]carfentanil and [^18^F]FMPEP-*d*_*2*_ PET images were acquired with PET/CT (GE Discovery VCT PET/CT, GE Healthcare). The tracer was administered in a catheter placed in subject’s antecubital vein. Subject’s head was strapped to the scan table to prevent excessive head movement. Computed tomography scans were acquired before PET scans for attenuation correction. The subjects were clinically monitored by physician throughout the scans. In the [^18^F]FDG and [^18^F]FMPEP-*d*_*2*_ scans, the plasma radioactivity was measured from arterialized blood samples in fixed time intervals using automatic γ-counter (Wizard 1480 3”, Wallac, Turku, Finland). Anatomical T1-weighted MR images (TR, 8.1 ms; TE, 3.7 ms; flip angle, 7°; scan time, 263 s; 1 mm^3^ isotropic voxels) were obtained with PET/MR (Ingenuity TF PET/MR, Philips) for anatomical normalization and reference. Hyperinsulinemic euglycemic clamp was applied during the [^18^F]FDG scans as previously described [30].

### Image processing and modeling

Automated processing tool Magia [31] (https://github.com/tkkarjal/magia) was used to process the PET data. Processing began with motion-correction of the PET data followed by coregistration of the PET and MR images. Magia uses FreeSurfer (http://surfer.nmr.mgh.harvard.edu/) to define the regions of interest (ROIs) as well as the reference regions (here applicable to [^11^C]carfentanil data). The ROI-wise kinetic modeling was based on extraction of ROI-wise time-activity curves. Prior to calculation of parametric images, the [^18^F]FMPEP-*d*_*2*_ and [^11^C]carfentanil PET images were smoothed using Gaussian kernel to increase signal-to-noise ratio before model fitting (FWHM = 6 mm for [^18^F]FMPEP-*d*_*2*_, 2 mm for [^11^C]carfentanil). Parametric images were spatially normalized to MNI-space and finally smoothed using a Gaussian kernel (FWHM = 8 mm for [^18^F]FDG, 6 mm for [^11^C]carfentanil and [^18^F]FMPEP-*d*_*2*_). BGU-estimates (μmol/min/100g) obtained from the [^18^F]FDG PET data are based on fractional uptake rate [32]. [^11^C]carfentanil binding was quantified by binding potential (*BP*_ND_), which is the ratio of specific binding to nondisplaceable binding in the tissue [33]. Occipital cortex was used as the reference region [34]. CB_1_R availability was quantified as [^18^F]FMPEP-*d*_*2*_ volume of distribution (*V*_T_) using graphical analysis (Logan) [35]. The starting point of 36 min was used, since Logan plots became linear after 36 min from injection [35]. Detailed description about the modeling of each tracer is presented in Supplementary Text 1.

### Analysis of serum endocannabinoids

Circulating endocannabinoids might affect central CB_1_R availability [36]. Serum endocannabinoids and related fatty acids were analyzed from fasting-state blood samples drawn in [^18^F]FMPEP-*d*_2_ scan day as described previously [36], with slight modifications (see Supplementary Text 2 for the full description). Serum endocannabinoid levels are shown in Supplementary Table 1.

### Experimental design and statistical analysis

#### Primary analyses

The primary outcome variables in the analyses were BGU measured with [^18^F]FDG, [^11^C]carfentanil *BP*_ND_ and [^18^F]FMPEP-*d*_*2*_ *V*_T_. The primary study question was whether these outcome variables differ between the LR and HR groups. Full-volume data were analyzed with nonparametric testing using SnPM13 (http://nisox.org/Software/SnPM13/). We used *p* < 0.05 as the cluster-defining threshold, and only report clusters large enough to be statistically significant (FWE *p* < 0.05). A total of 5000 permutations were used to estimate the null distribution. LR and HR groups were compared using two-sample t-test. Age was included as a covariate in all full-volume models, since age is known to affect at least [^11^C]carfentanil binding [37, 38] and [^18^F]FDG uptake [39].

#### Secondary analyses

Additionally, we analyzed the associations of individual risk factors (BMI, physical exercise and Family Risk) to the PET outcome variables (BGU, *BP*_ND_, and *V*_T_) in a priori ROIs with Bayesian approach. Based on previous studies [12, 40, 41], FreeSurfer (http://surfer.nmr.mgh.harvard.edu/) was used to extract 21 bilateral ROIs involved in emotion and food reward processing: amygdala, caudate, cerebellum, dorsal anterior cingulate cortex, hippocampus, inferior temporal gyrus, insula, medulla, midbrain, middle temporal gyrus, nucleus accumbens, orbitofrontal cortex, pars opercularis, posterior cingulate cortex, pons, putamen, rostral anterior cingulate cortex, superior frontal gyrus, superior temporal gyrus, temporal pole, and thalamus. We used varying (random) slopes and intercepts for the ROIs, and thus the results do not require separate correction for multiple ROIs [42]. We used regularizing priors (zero-mean normal distribution with unit-variance) for the regression coefficients to reduce overfitting. Bayesian hierarchical modeling was done with the R package BRMS (https://cran.r-project.org/package=brms) that uses the efficient Markov chain Monte Carlo sampling tools of RStan (https://mc-stan.org/users/interfaces/rstan). We fitted the models separately for body mass index, familial obesity risk, and physical exercise. All models also included age as a nuisance covariate. We used weakly informative priors: For intercepts, we used the default of BRMS, i.e., Student’s *t* distribution with scale 3 and 10 degrees of freedom. For predictors, a Gaussian distribution with standard deviation of 1 was used to provide weak regularization. The BRMS default prior half Student’s *t* distribution with 3 degrees of freedom was used for standard deviations of group-level effects; BRMS automatically selects the scale parameter to improve convergence and sampling efficiency. The BRMS default prior LKJ(1) was used for correlations of group-level random effects. The ROI-level models were estimated using five chains, each of which had 1000 warmup samples and 4000 post-warmup samples, thus totaling 20000 post-warmup samples. The sampling parameters were slightly modified to facilitate convergence (adapt_delta = 0.99; max_treedepth = 20). The sampling produced no divergent iterations and the Rhats were all 1.0, suggesting that the chains converged successfully. Before model estimation, continuous predictors were standardized to have zero mean and unit variance, thus making the regression coefficients comparable across the predictors. All outcome variables ([^11^C]carfentanil *BP*_ND_, [^18^F]FMPEP-*d*_*2*_ *V*_T_ and BGU) were log-transformed to improve model fit [37]. Since all three outcome variables had associations with the Family Risk score, we created *post hoc* Bayesian linear regression model with age-adjusted BGU, *BP*_ND_ and *V*_T_ values as the predictors of Family Risk to assess the relative effects of the outcome variables.

In addition, serum endocannabinoids were studied in separate full volume models of [^18^F]FMPEP-*d*_*2*_ *V*_T_. Since eight distinct endocannabinoid compounds were analyzed, we confirmed the results with Bonferroni-corrected p value as the cluster-defining threshold (0.05/8 = 0.00625).

## Results

**Fig. 1:**
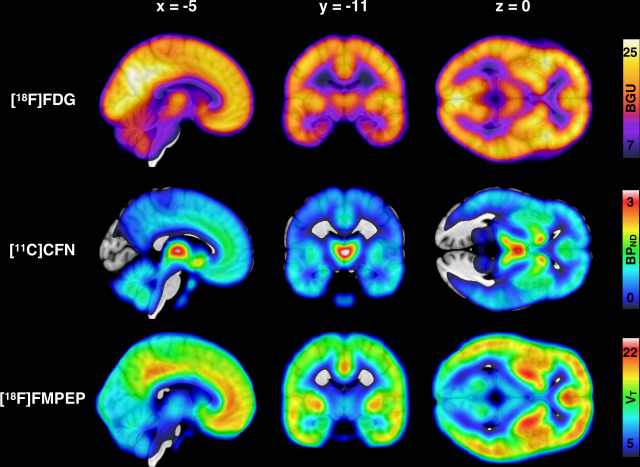
Mean distribution of brain glucose uptake, μ-opioid receptor availability and CB_1_ receptor availability in the whole study sample. Top: Mean brain glucose uptake (BGU) of the 38 [^18^F]FDG scans (19 low-risk and 19 high-risk subjects). Middle: Mean binding potential (*BP*_ND_) of the 41 [^11^C]carfentanil scans (22 low-risk and 19 high-risk subjects). Bottom: Mean volume of distribution (*V*_T_) of the 36 [^18^F]FMPEP-*d*_*2*_ scans (20 low-risk and 16 high-risk subjects).

Mean distribution of BGU, MOR availability and CB_1_R availability are shown in Fig. 1. Descriptive Pearson correlations of the sample are presented in the Supplementary Fig. 1 and Supplementary Table 2.

### Risk group comparisons

**Fig. 2:**
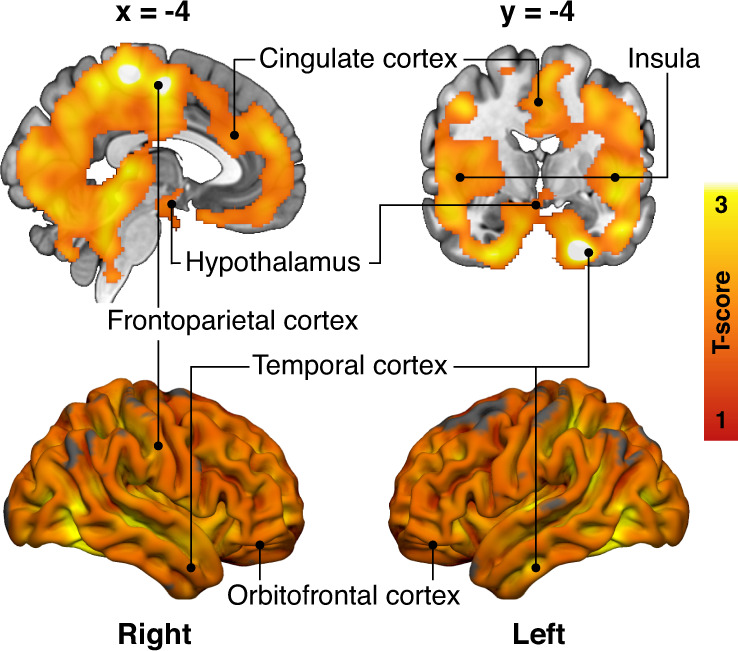
Obesity risk and brain glucose uptake. Brain regions where the high-risk subjects (*n* = 19) had increased brain glucose uptake (BGU) compared with the low-risk subjects (*n* = 19) while controlling for age. The data are thresholded at *p* < 0.05, FWE corrected at cluster level. T-score from the two-sample t-test is shown in red-to-yellow scale.

HR group had increased BGU compared to the LR group in multiple brain regions. Prominent associations were found in frontotemporal and cingulate cortices, hypothalamus, and bilaterally in insula and putamen (Fig. 2). MOR or CB_1_R availabilities did not have statistically significant differences between the two groups.

### Effects of distinct risk factors

#### Brain glucose uptake

**Fig. 3:**
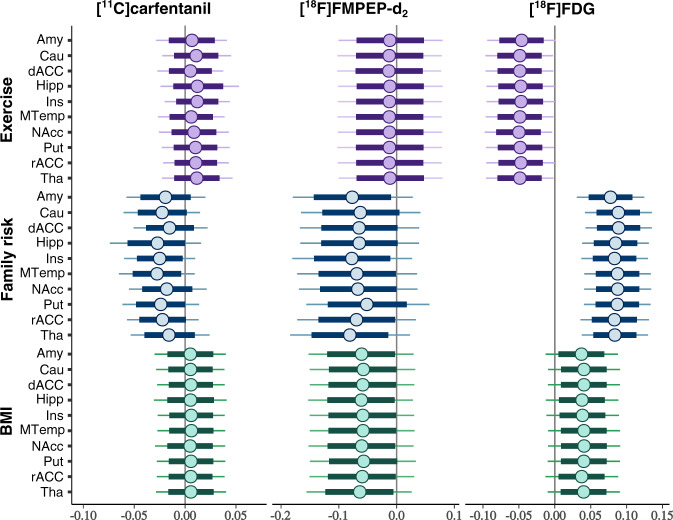
Effects of obesity risk factors on brain glucose uptake and neuroreceptor availability in ten representative regions of interest. Posterior distributions of the regression coefficients for exercise, family risk and body mass index (BMI) on log-transformed binding potential (*BP*_ND_) of the [^11^C]carfentanil, volume of distribution (*V*_T_) of the [^18^F]FMPEP-*d*_*2*_ and brain glucose uptake (BGU) quantified with [^18^F]FDG in representative regions of interest, age as a covariate. The colored circles represent posterior means, the thick horizontal bars 80% posterior intervals, and the thin horizontal bars 95% posterior intervals. The width of posterior intervals illustrates the level of uncertainty of the estimate. Abbreviations: Amy = amygdala, Cau = caudate, dACC = dorsal anterior cingulate cortex, Hipp = hippocampus, Ins = insula, MTemp = middle temporal gyrus, NAcc = nucleus accumbens, Put = putamen, rACC = rostral anterior cingulate cortex, Tha = thalamus.

**Fig. 4:**
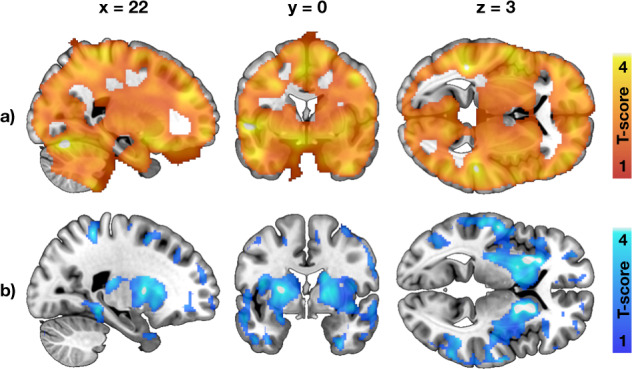
Effects of familial obesity risk to central glucose uptake and μ-opioid receptor availability. **a** Brain regions where higher Family Risk score associated with increased brain glucose uptake in the 38 individuals studied with [^18^F]FDG. **b** Brain regions where higher Family Risk score associated with lower μ-opioid receptor availability in the 41 individuals studied with [^11^C]carfentanil. The effects of familial obesity risk we global for brain glucose uptake, whereas the associations were most prominent in striatum and insula for μ-opioid receptors. The images show results from SnPM13 linear regression, with age and other risk factors (BMI, physical exercise) as covariates. The data are thresholded at *p* < 0.05, FWE corrected at cluster level.

Increase in Family Risk was associated with globally increased in BGU (Fig. 3, all ROIs in Supplementary Fig. 2). BMI had a moderate positive association with BGU, while increased physical exercise associated with lower BGU (Fig. 3). Full volume visualization of the Family Risk associations is presented in Fig. 4.

#### μ-opioid receptor availability

Higher Family Risk was associated with lower *BP*_ND_ in frontotemporal cortex, insula and striatum (Figs. 3 and 4, all ROIs in Supplementary Fig. 2), while the effects of BMI and physical exercise markedly overlapped with zero.

#### CB_1_ receptor availability

Family Risk and BMI had negative association with *V*_T_ (Fig. 3, all ROIs in Supplementary Fig. 2). Anandamide (AEA) was the only endocannabinoid to exhibit significant effects to *V*_T_. Increase in serum AEA was associated with lower *V*_T_ in frontal striatum (Supplementary Fig. 3).

#### Linear regression analysis of family risk with three tracers

Finally, we pooled the data across the three radioligands in a reversed analysis to test which cerebral alterations are the best predictors of familial obesity risk. Increased BGU explained the higher Family Risk score in every ROI. The posterior distributions of [^11^C]carfentanil *BP*_ND_ and [^18^F]FMPEP-*d*_*2*_ *V*_T_ mostly overlapped with zero, and the directions of the associations were negative (Supplementary Fig. 4).

## Discussion

Our main finding was that non-obese young males with high risk for future obesity had increased insulin-stimulated brain glucose uptake. Furthermore, the increased familial obesity risk (i.e., parental obesity and T2D prevalence) was associated with lowered μ-opioid and CB_1_ receptor density in addition to the globally altered glucose metabolism in the brain. Brain glucose uptake had the strongest and most consistent association with familial obesity risk among the three examined PET variables.

Sedentary lifestyle combined to readily available high-calorie food has been proposed to be core issue for obesity epidemic [43], yet hereditary factors play a key role in individual obesity risk. Multiple genes contribute to susceptibility for obesity and T2D, and a first-degree relative with obesity raises individual’s obesity risk two- to threefold [44]. Our results highlight that molecular and metabolic alterations in the brain are also associated with individual obesity risk. The present findings complement those previously reported in middle-aged obese subjects, suggesting that alterations in brain’s insulin signaling and MOR and CB_1_R neurotransmission might contribute to elevated risk for gaining weight.

### Brain glucose uptake and obesity risk

The non-obese subjects with high risk for developing obesity showed widespread increase in brain glucose uptake during hyperinsulinemia—a phenomenon that has been previously reported in morbidly obese individuals [12]. It has been suggested that central inflammation, impaired insulin transport regulation in the blood-brain barrier and diminished neuronal responses to insulin might promote these changes in fully developed obesity [12, 45, 46]. The present results show that these pathophysiological processes are active already in non-obese subjects with risk factors for obesity: HR group had globally increased BGU compared to the LR group, and a familial history of obesity and T2D were strongly linked to increased BGU. Central insulin resistance has been proposed to underlie the pathogenesis of obesity and T2D [47]. Disturbances in brain insulin action and impaired signaling between the brain and peripheral organs may contribute to pathological energy homeostasis and weight gain. Familial obesity risk had positive correlation with BGU extensively in the brain, also in cingulate cortex, striatum and nucleus accumbens that have important role in the central regulation of food intake and reward [48]. Impaired insulin action in these brain regions, together with altered neuroreceptor signaling, could potentially predispose to increased food intake and weight gain.

### Central μ-opioid receptor downregulation as an obesity-promoting mechanism

Parental obesity and T2D were associated with lower MOR availability in non-obese state in multiple brain areas, including frontal cortex, striatum, and insula. Previously MOR downregulation in same brain areas has been found in patients with morbid obesity [23] and binge eating disorder (BED) [49]. These alterations accord with genetic studies suggesting that variability in MOR-coding gene OPRM1 is linked with MOR function and eating behavior. Variation in OPRM1 (SNP rs1799971, prevalence in Finland ∼19% [50]) reduces MOR availability [51] and is over-represented in patients with BED [52]. MOR system mediates feeding and reward [14], and prior studies have found that MOR downregulation makes an individual more sensitive to environment’s rewarding food cues [53]. Individuals with hereditary predisposition towards downregulated MORs may thus be more sensitive to respond to the anticipatory food cues in the environment, leading to excess feeding [54]. Alternatively, they might also compensate the reduced MOR availability by excessive food intake to get sufficient reward response and incentive to halt food intake. These proposed mechanisms could possibly lead to vicious cycle in feeding behavior, where excessive endogenous opioid stimulation by feeding [55] would cause further MOR downregulation and vice versa [16].

### CB_1_ receptor availability and endogenous cannabinoids

Our results suggest that higher familial obesity risk and higher body mass are associated with lower CB_1_R availability in the brain. Our findings are in line with a prior PET study that has linked increase in BMI to lower CB_1_R availability [41]. In a recent PET study, serum endocannabinoid peptides (including AEA) had negative relationship with central CB_1_R availability [36]. Our results add support to these earlier findings: we found that serum AEA concentration had negative relationship with CB_1_R in ventral striatum already in the non-obese state. In animal studies, AEA has been shown to stimulate food intake via activation of central CB_1_Rs [56] and to amplify hedonic reward responses to sweet taste [57]. In obese humans, serum AEA is increased and associates with decreased CB_1_R gene expression [58]. The elevated systemic AEA concentration might thus be a pathophysiological trait promoting CB_1_R downregulation, and possibly weight gain.

### Limitations and future directions

First, since we studied only males, the conclusions may not be generalizable to females. Second, there was a small age-difference between the two risk groups. Age was however included in all analyses as a nuisance covariate. By study design, the BMI of HR group was higher than the BMI in LR group, since overweight in early adulthood is a predictive factor for future obesity [4]. However, compared to BMI, familial obesity risk had generally stronger and independent effects to the brain glucose uptake and neuroreceptor availability (Figs. 3 and 4). Also in healthy males, BMI in the range of 18–34 does not affect central MOR availability [37]. Third, we did not have the information about genetic profile of the subjects, and were therefore not able to directly assess the genetic obesity risk. Fourth, the information about physical exercise and parental risk factors were acquired by interview by licensed physician with a standardized medical history checkup, rather than by direct measurement. Finally, as a cross-sectional work this study cannot differentiate whether the detected cerebral alterations are the cause or the effect of increasing obesity risk or whether one receptor system’s alteration would serve as the primal cause for the detected changes in the other systems. In a single PET scan, it is also not possible to demonstrate the exact molecule-level mechanism for altered receptor availability [37]. Follow-up studies with assessment of eating behavior are needed to confirm the proposed effects of these brain signaling alterations to future weight gain.

## Conclusions

Individuals with well-established risk factors for obesity have alterations in the brain’s insulin responsivity and opioid and endocannabinoid signaling that resemble those observed in obesity. History of parental obesity and T2D is manifested as altered cerebral insulin sensitivity and reduced MOR and CB_1_R availability. The detected neurochemical alterations emphasize the hereditary and centrally mediated mechanisms in obesity development. Disturbance of these integrative food intake control systems in the brain may potentially predispose to weight gain and obesity.

## Supplementary information

Supplementary Material

## Data Availability

The code for preprocessing of the PET data (Magia) is available at https://github.com/tkkarjal/magia.

## References

[1] Abarca-Gómez L, Abdeen ZA, Hamid ZA, Abu-Rmeileh NM, Acosta-Cazares B, Acuin C (2017). Worldwide trends in body-mass index, underweight, overweight, and obesity from 1975 to 2016: a pooled analysis of 2416 population-based measurement studies in 128·9 million children, adolescents, and adults. Lancet.

[2] Van Gaal LF, Mertens IL, De, Block CE (2006). Mechanisms linking obesity with cardiovascular disease. Nature.

[3] Kivipelto M, Ngandu T, Fratiglioni L, Viitanen M, Kareholt I, Winblad B (2005). Obesity and vascular risk factors at midlife and the risk of dementia and Alzheimer disease. Arch Neurol.

[4] Juhola J, Magnussen CG, Viikari JSA, Kähönen M, Hutri-Kähönen N, Jula A (2011). Tracking of serum lipid levels, blood pressure, and body mass index from childhood to adulthood: the cardiovascular risk in Young Finns study. J Pediatr.

[5] Parsons TJ, Power C, Logan S, Summerbell CD (1999). Childhood predictors of adult obesity: a systematic review. Int J Obes Relat Metab Disord.

[6] Haffner SM, Stern MP, Hazuda HP, Mitchell BD, Patterson JK, Ferrannini E (1989). Parental history of diabetes is associated with increased cardiovascular risk factors. Arteriosclerosis.

[7] Anjana RM, Lakshminarayanan S, Deepa M, Farooq S, Pradeepa R, Mohan V (2009). Parental history of type 2 diabetes mellitus, metabolic syndrome, and cardiometabolic risk factors in Asian Indian adolescents. Metabolism.

[8] Yang X, Telama R, Leskinen E, Mansikkaniemi K, Viikari J, Raitakari OT (2007). Testing a model of physical activity and obesity tracking from youth to adulthood: the cardiovascular risk in young Finns study. Int J Obes.

[9] Juonala M, Juhola J, Magnussen CG, Würtz P, Viikari JSA, Thomson R (2011). Childhood environmental and genetic predictors of adulthood obesity: the cardiovascular risk in Young Finns study. J Clin Endocrinol Metab.

[10] Guyenet SJ, Schwartz MW (2012). Regulation of food intake, energy balance, and body fat mass: implications for the pathogenesis and treatment of obesity. J Clin Endocrinol Metab.

[11] Davis JF, Choi DL, Benoit SC (2010). Insulin, leptin and reward. Trends Endocrinol Metabol.

[12] Tuulari JJ, Karlsson HK, Hirvonen J, Hannukainen JC, Bucci M, Helmio M (2013). Weight loss after bariatric surgery reverses insulin-induced increases in brain glucose metabolism of the morbidly obese. Diabetes.

[13] Anthony K, Reed LJ, Dunn JT, Bingham E, Hopkins D, Marsden PK (2006). Attenuation of insulin-evoked responses in brain networks controlling appetite and reward in insulin resistance: the cerebral basis for impaired control of food intake in metabolic syndrome?. Diabetes.

[14] Gosnell BA, Levine AS (2009). Reward systems and food intake: role of opioids. Int J Obes.

[15] Yeomans MR, Gray RW (2002). Opioid peptides and the control of human ingestive behaviour. Neurosci Biobehav Rev.

[16] Karlsson HK, Tuulari JJ, Tuominen L, Hirvonen J, Honka H, Parkkola R (2016). Weight loss after bariatric surgery normalizes brain opioid receptors in morbid obesity. Mol Psychiatry.

[17] Bermudez-Silva FJ, Cardinal P, Cota D (2012). The role of the endocannabinoid system in the neuroendocrine regulation of energy balance. J Psychopharmacol.

[18] Mechoulam R, Parker LA (2013). The endocannabinoid system and the brain. Annu Rev Psychol.

[19] Kirkham TC, Williams CM, Fezza F, Marzo VD (2002). Endocannabinoid levels in rat limbic forebrain and hypothalamus in relation to fasting, feeding and satiation: stimulation of eating by 2-arachidonoyl glycerol. Br J Pharmacol.

[20] Ravinet Trillou C, Delgorge C, Menet C, Arnone M, Soubrié P (2004). CB1 cannabinoid receptor knockout in mice leads to leanness, resistance to diet-induced obesity and enhanced leptin sensitivity. Int J Obes.

[21] Sanchis-Segura C, Cline BH, Marsicano G, Lutz B, Spanagel R (2004). Reduced sensitivity to reward in CB1 knockout mice. Psychopharmacology.

[22] Harrold JA, Elliott JC, King PJ, Widdowson PS, Williams G (2002). Down-regulation of cannabinoid-1 (CB-1) receptors in specific extrahypothalamic regions of rats with dietary obesity: a role for endogenous cannabinoids in driving appetite for palatable food?. Brain Res.

[23] Karlsson HK, Tuominen L, Tuulari JJ, Hirvonen J, Parkkola R, Helin S (2015). Obesity is associated with decreased mu-opioid but unaltered dopamine D2 receptor availability in the brain. J Neurosci.

[24] Hamacher K, Coenen HH, Stöcklin G (1986). Efficient stereospecific synthesis of no-carrier-added 2-[18F]-fluoro-2-deoxy-D-glucose using aminopolyether supported nucleophilic substitution. J Nucl Med.

[25] Lemaire C, Damhaut P, Lauricella B, Mosdzianowski C, Morelle JL, Monclus M (2002). Fast [18F]FDG synthesis by alkaline hydrolysis on a low polarity solid phase support. J Label Compd Radiopharm.

[26] Eriksson O, Antoni G (2015). [11C]Carfentanil binds preferentially to mu-opioid receptor subtype 1 compared to subtype 2. Mol Imaging.

[27] Larsen P, Ulin J, Dahlstrøm K, Jensen M (1997). Synthesis of [11C] iodomethane by iodination of [11C] methane. Appl Radiat Isot.

[28] Jewett DM (1992). A simple synthesis of [11C] methyl triflate. Int J Radiat Appl Instrum.

[29] Lahdenpohja S, Keller T, Forsback S, Viljanen T, Kokkomäki E, Kivelä RV, et al. Automated GMP production and long‐term experience in radiosynthesis of CB1 tracer [18F] FMPEP‐d2. J Label Compd Radiopharm. 2020;63:408–18. 10.1002/jlcr.3845 32374481

[30] DeFronzo RA, Tobin JD, Andres R (1979). Glucose clamp technique: a method for quantifying insulin secretion and resistance. Am J Physiol-Endocrinol Metab.

[31] Karjalainen T, Tuisku J, Santavirta S, Kantonen T, Bucci M, Tuominen L, et al. Magia: robust automated image processing and kinetic modeling toolbox for PET neuroinformatics. Front Neuroinform*.* 2020;14:3. 10.3389/fninf.2020.00003 PMC7012016 32116627

[32] Thie JA (1995). Clarification of a fractional uptake concept. J Nucl Med.

[33] Innis RB, Cunningham VJ, Delforge J, Fujita M, Gjedde A, Gunn RN (2007). Consensus nomenclature for in vivo imaging of reversibly binding radioligands. J Cereb Blood Flow Metab.

[34] Frost JJ, Douglass KH, Mayberg HS, Dannals RF, Links JM, Wilson AA (1989). Multicompartmental analysis of [11C]-Carfentanil binding to opiate receptors in humans measured by positron emission tomography. J Cereb Blood Flow Metab.

[35] Logan J (2000). Graphical analysis of PET data applied to reversible and irreversible tracers. Nuclear Med Biol.

[36] Dickens AM, Borgan F, Laurikainen H, Lamichhane S, Marques T, Rönkkö T (2020). Links between central CB1-receptor availability and peripheral endocannabinoids in patients with first episode psychosis. npj Schizophr.

[37] Kantonen T, Karjalainen T, Isojärvi J, Nuutila P, Tuisku J, Rinne J (2020). Interindividual variability and lateralization of μ-opioid receptors in the human brain. NeuroImage.

[38] Zubieta JK, Dannals RF, Frost JJ (1999). Gender and age influences on human brain mu-opioid receptor binding measured by PET. Am J Psychiatry.

[39] Loessner A, Alavi A, Lewandrowski K-U, Mozley D, Souder E, Gur R (1995). Regional cerebral function determined by FDG-PET in healthy volunteers: normal patterns and changes with age. J Nucl Med.

[40] Tuominen L, Nummenmaa L, Keltikangas‐Järvinen L, Raitakari O, Hietala J (2014). Mapping neurotransmitter networks with PET: an example on serotonin and opioid systems. Hum Brain Mapp.

[41] Hirvonen J, Goodwin RS, Li CT, Terry GE, Zoghbi SS, Morse C (2012). Reversible and regionally selective downregulation of brain cannabinoid CB1 receptors in chronic daily cannabis smokers. Mol Psychiatry.

[42] Gelman A, Hill J, Yajima M (2012). Why we (usually) don’t have to worry about multiple comparisons. J Res Educ Effect.

[43] Hill JO, Peters JC (1998). Environmental contributions to the obesity epidemic. Science.

[44] Loos RJ, Bouchard C (2003). Obesity—is it a genetic disorder?. J Intern Med.

[45] Hallschmid M, Schultes B (2009). Central nervous insulin resistance: a promising target in the treatment of metabolic and cognitive disorders?. Diabetologia.

[46] Rebelos E, Rinne JO, Nuutila P, Ekblad LL (2021). Brain glucose metabolism in health, obesity, and cognitive decline—does insulin have anything to do with it? A narrative review. J Clin Med.

[47] Kullmann S, Heni M, Hallschmid M, Fritsche A, Preissl H, Haring HU (2016). Brain insulin resistance at the crossroads of metabolic and cognitive disorders in humans. Physiol Rev.

[48] Stoeckel LE, Weller RE, Cook EW, Twieg DB, Knowlton RC, Cox JE (2008). Widespread reward-system activation in obese women in response to pictures of high-calorie foods. Neuroimage.

[49] Majuri J, Joutsa J, Johansson J, Voon V, Alakurtti K, Parkkola R (2017). Dopamine and opioid neurotransmission in behavioral addictions: a comparative PET study in pathological gambling and binge eating. Neuropsychopharmacology.

[50] Rouvinen-Lagerström N, Lahti J, Alho H, Kovanen L, Aalto M, Partonen T (2013). µ-Opioid receptor gene (OPRM1) polymorphism A118G: lack of association in Finnish populations with alcohol dependence or alcohol consumption. Alcohol Alcohol.

[51] Weerts EM, McCaul ME, Kuwabara H, Yang X, Xu X, Dannals RF (2013). Influence of OPRM1 Asn40Asp variant (A118G) on [11C]carfentanil binding potential: preliminary findings in human subjects. Int J Neuropsychopharmacol.

[52] Davis CA, Levitan RD, Reid C, Carter JC, Kaplan AS, Patte KA (2009). Dopamine for “wanting” and opioids for “liking”: a comparison of obese adults with and without binge eating. Obesity.

[53] Nummenmaa L, Saanijoki T, Tuominen L, Hirvonen J, Tuulari JJ, Nuutila P (2018). μ-opioid receptor system mediates reward processing in humans. Nat Commun.

[54] Kantonen T, Karjalainen T, Pekkarinen L, Isojärvi J, Kalliokoski K, Kaasinen V (2021). Cerebral μ-opioid and CB1 receptor systems have distinct roles in human feeding behavior. Transl Psychiatry.

[55] Tuulari JJ, Tuominen L, de Boer FE, Hirvonen J, Helin S, Nuutila P (2017). Feeding releases endogenous opioids in humans. J Neurosci.

[56] Jamshidi N, Taylor DA (2001). Anandamide administration into the ventromedial hypothalamus stimulates appetite in rats. Br J Pharmacol.

[57] Mahler SV, Smith KS, Berridge KC (2007). Endocannabinoid hedonic hotspot for sensory pleasure: anandamide in nucleus accumbens shell enhances ‘liking’ of a sweet reward. Neuropsychopharmacology.

[58] Engeli S, Böhnke J, Feldpausch M, Gorzelniak K, Janke J, Bátkai S (2005). Activation of the peripheral endocannabinoid system in human obesity. Diabetes.

