# Supplementary material for: Obesity risk is associated with altered cerebral glucose metabolism and decreased μ-opioid and CB1 receptor availability
Source: Int J Obes (Lond). 2021 Nov 2;46(2):400–7. doi: 10.1038/s41366-021-00996-y (PMC8794779; doi:10.1038/s41366-021-00996-y)
Supplement: Supplementary file 1 — Supplementary Material [file 41366_2021_996_MOESM1_ESM.pdf]

Supplementary Material

i. Detailed scan protocols, hyperinsulinemic euglycemic clamp execution and image modeling

ii. Analysis of serum endocannabinoids

iii. Serum endocannabinoid levels

iv. Table of the p-values for descriptive correlations

v. Descriptive correlations of the sample

vi. Regional effects obesity risk factors

vii. Serum anandamide and CB_1 receptor availability

viii. Linear regression analysis of family risk with three tracers

Detailed scan protocols, hyperinsulinemic euglycemic clamp execution and image modeling

Supplementary Text 1. Detailed scan protocols for [^{11}C]carfentanil, [^{18}F]FDG, [^{18}F]FMPEP-*d_2* scans, hyperinsulinemic euglycemic clamp execution and image modeling.

[^{11}C]carfentanil scans

A cannula was inserted before the scan in an antecubital vein for [^{11}C]carfentanil injection. 223–279 MBq of [^{11}C]carfentanil was administered intravenously and the brain's radioactivity was followed 51 minutes (13 frames).

[^{18}F]FDG scans and hyperinsulinemic euglycemic clamp studies

Two cannulas were inserted before the scan, one in an antecubital vein for infusion of insulin, glucose and [^{18}F]FDG injection and one in the contralateral antecubital vein for blood sampling. 130–175 MBq of [^{18}F]FDG was administered intravenously and a peripheral scan for thoracic region (40 min), abdomen (15 min), thighs (15 min) and neck (10 min) was conducted before the brain scan. Brain scan started 90 ± 2 min after the injection and lasted 10 minutes. To measure plasma activity, arterialized venous blood samples were drawn contralaterally to injection site at 4.5, 7.5, 10, 20 and 30 min from the injection, and in the middle time points of the abdomen, thighs, neck and brain scans.

The [^{18}F]FDG scan was performed during an hyperinsulinemic euglycemic clamp, applied as previously described.[1] Briefly, subjects were administered intravenous insulin (Actrapid, Novo Nordisk A/S, Bagsvaerd, Denmark) at a steady rate of 1 mU/kg/min throughout the study. Plasma glucose measurements were taken every 5 to 10 minutes from arterialized venous blood, plasma glucose was maintained at 5 mmol/l (5.5 ± 0.7 mmol/l) by using a variable rate infusion of 20 % glucose solution. The clamp was started 80 ± 13 minutes before the [^{18}F]FDG injection in order to reach steady glycemia before the PET scan and was continued throughout the PET study.

[^{18}F]FMPEP-d_2 scans

Two cannulas were inserted before the scan, one in an antecubital vein for [^{18}F]FMPEP-d_2 injection and one in the contralateral antecubital vein for blood sampling. Before the scan, venous blood samples were drawn to measure hematocrit and serum endocannabinoid levels. 147–215 MBq of [^{18}F]FMPEP-d_2 was administered intravenously and brain's radioactivity was followed 60 minutes (15 frames). A peripheral PET scan was conducted on neck (12 min) and abdominal (9 min) regions. Brain's radioactivity was then followed 9 minutes (3 x 180-second frames) starting at a time point 94 ± 2 min. Arterialized venous blood samples to measure plasma activity were drawn contralaterally to injection site at 0.25, 0.5, 0.75, 1, 1.25, 1.5, 1.75, 2, 2.5, 3, 4.5, 7.5, 11, 15, 20, 25, 30, 35, 40, 45, 50 and 60 min from the injection, and at subsequent time points (min from the regional scan start): neck (2 and 6 min), abdomen (4.5 min) and late brain scan (4.5 min). Additional blood samples for [^{18}F]FMPEP-d_2 metabolite analysis were drawn before the scan and at 4.5, 11, 15, 20, 30, 45 and 60 min from the injection, and at subsequent time points (min from the regional scan start): neck (2 and 6 min), abdomen (4.5 min).

Image modeling

The [^{11}C]carfentanil binding was quantified by binding potential (BP_{ND}), which is the ratio of specific binding to non-displaceable binding in the tissue.[2] Simplified reference tissue model[3] was used to estimate BP_{ND}. Occipital cortex served as the reference region.[4]

CB_1R availability was quantified as [^{18}F]FMPEP-d_2 volume of distribution (V_{T}) using graphical analysis[5] (Logan). The frames starting at 36 minutes and later since injection were used in the model fitting, since Logan plots became linear after 36 minutes.[5] Plasma activities were corrected for plasma metabolites as described previously.[6] We were unable to obtain metabolite data from two LR subjects due to laboratory measurement issues, so we used the mean metabolite fraction of the LR group for these subjects.

BGU-estimates obtained from the [^{18}F]FDG PET data are based on FUR.[7] The input functions were hybrid-functions – from 0 to 4.5 min we used image-derived inputs from the left ventricle and

combined them to the arterialized plasma sampling derived inputs from 4.5 min to the end of the scan. The input functions were inspected for quality control, using a selection criterion (good/poor quality) based on a linear regression including the maximum peak of the input functions (Standardized Uptake Value curves) and the ratio peak/5th min of the input time activity curve. The selection criterion has been validated against reference input curves of good quality. According to this selection criterion, two inputs (of LR subjects) were not acceptable for analysis. It was possible to recover the inputs of bad quality using an input recovery method that utilizes the tail of the poor quality curves (from the 5th min till the end) to estimate curves from a trained estimation model, validated on reference (mainly) arterial input curves. In brief, the input estimation model is based on the Feng input equation fitting,[8] mixed with a Bayesian approach via the use of constraints derived by linear regressions of modeling parameters and characteristics of the reference curves. Fitted parameter averages of the Feng function of the reference curves are also used as one of the elements of the Bayesian minimization algorithm.

The FUR-values were converted into BGU-estimates using the equation

(1) $BGU = 100\frac{[glucose]*FUR}{LC*density}$

where [glucose] is the average glucose concentration in the blood from the time of the tracer [^{18}F]FDG injection till the end of the brain scan, LC is the lumped constant, and density is the gray matter tissue relative density in the brain. We used values LC = 0.65[9] and density = 1.04[10] in the calculations.

Analysis of serum endocannabinoids

Supplementary Text 2. Analysis of serum endocannabinoids and related fatty acids.

The analysis of serum endocannabinoids was based on a previously reported method,[11] with the addition of N-arachidonoyl-L-serine (NALS), N-arachidonoyl taurine (NAT), docosatetraenoyl ethanolamide (DEA), alpha-linolenoyl ethanolamide (αLEA), and gamma-linolenoyl ethanolamide (γLEA). The sample preparation steps were performed automatically using a Microlab STAR system (Hamilton, Reno, NV), and protein centrifugation was replaced by filtration using 96-well protein precipitation filter plates (Supelco, Bellefonte, PA). The endocannabinoids were separated using ultra-high-performance liquid chromatography (UHPLC) ExionLC AD (Sciex Inc., Framingham, MA). The two eluent solvents were (i) H_2O (1% v/v acetic acid) and (ii) ACN. The gradient was set up as follows: the initial conditions were 55% B increasing to 60% by 3 minutes, then increased to 70% B by 6 minutes, to 80% B by 8 minutes and held in these conditions for 3 minutes. Finally, the gradient was reduced to starting conditions by 11.1 minutes, and the column flushed with a minimum of 10 column volumes. The flow rate was set at 0.5 ml/min throughout the run and the column temperature set to 60 °C. The column used for the separation was a Waters ACQUITY BEH reverse phase C18 column (130 Å, 1.7 µm, 2.1 mm × 50 mm). The injection volume was 10 µl. The needle was washed three times both before and after the injection with two solvents: (i) IPA:ACN (1:1), and (ii) H_2O:MeOH (1:1). The mass spectrometer 6500+ QTRAP (Sciex Inc., Framingham, MA) was set up in scheduled multiple reaction monitoring (MRM) mode which allows for the setup of detection windows around the peak of interest to improve sensitivity.

Serum endocannabinoid levels

Supplementary Table 1. Serum endocannabinoid and related fatty acid levels of the 20 low-risk and 16 high-risk subjects that completed the [^{18}F]FMPEP-d_2 scan successfully. All endocannabinoid and fatty acid levels are reported in ng/ml. p-value is for two-tailed independent samples t-test between the two groups.

	Low-risk males (n = 20)		High-risk males (n = 16)		p-value
	mean	SD	mean	SD	
Arachidonic acid	388.5	132.4	428.2	111.5	0.34
Anandamide	0.23	0.07	0.28	0.10	0.08
Arachidonoyl glycerol (1 + 2)	3.7	1.4	4.5	2.2	0.15
α-linolenic acid	0.07	0.01	0.07	0.01	0.67
γ-linolenic acid	0.14	0.01	0.14	0.01	0.97
Docosatetraenoyl ethanolamide	0.01	0.01	0.02	0.02	0.18
N-arachidonoyl-L-serine	0.06	0.02	0.06	0.01	0.87
Oleyl ethanolamide	0.65	0.23	0.67	0.24	0.81

Table of the p-values for descriptive correlations

Supplementary Table 2. The p-values for corresponding Pearson correlation coefficients. Correlations are calculated with the 34 subjects (18 low-risk and 16 high risk subjects) that had no data points missing.

	Age	BMI	Exercise	Familyrisk
Age	1.000	0.038	0.043	<0.001
BMI	0.038	1.000	0.009	<0.001
Exercise	0.043	0.009	1.000	0.002
Familyrisk	<0.001	<0.001	0.002	1.000

Descriptive correlations of the sample

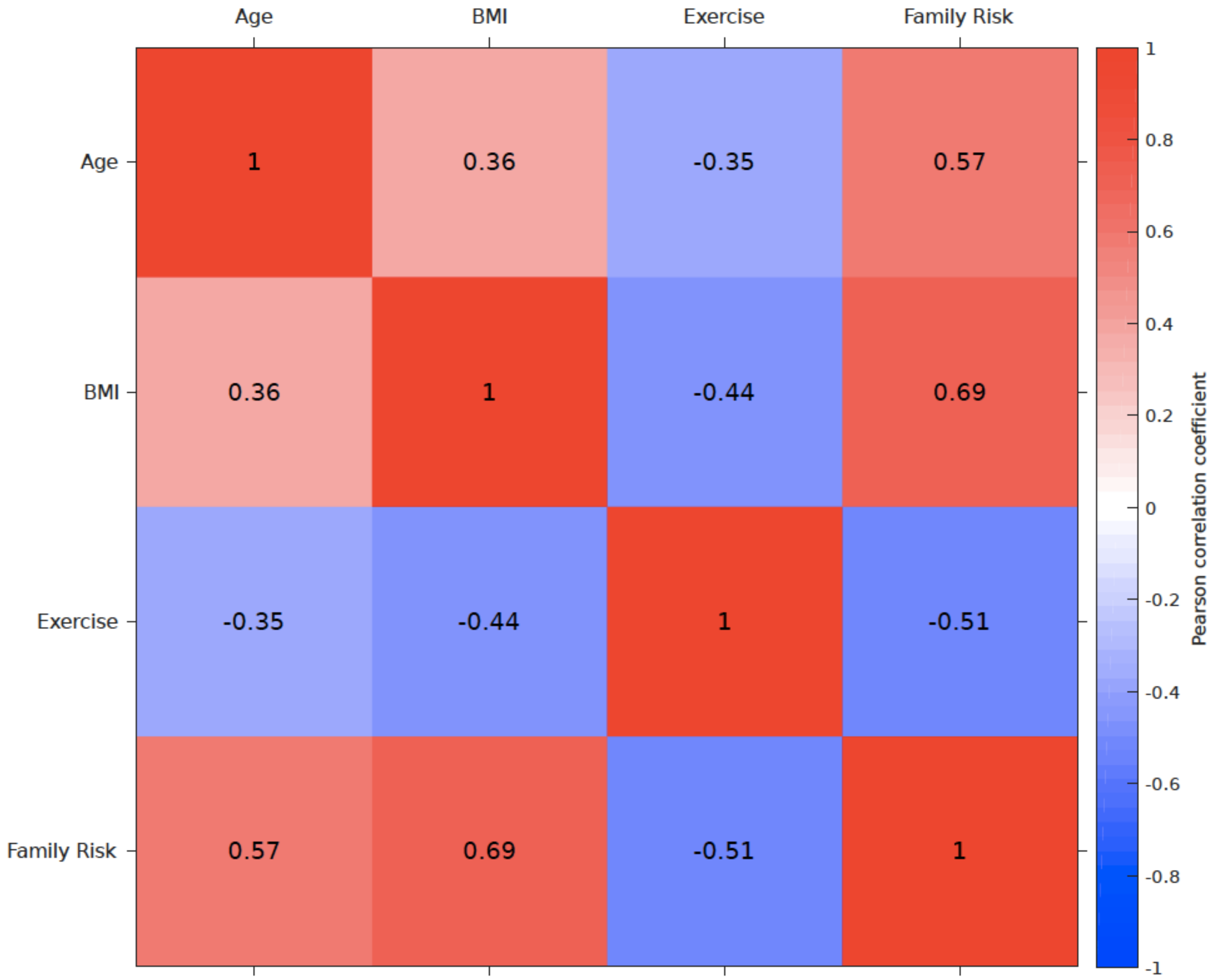

Supplementary Figure 1. Pearson correlation coefficients between sample describing variables. Correlations are calculated with the 34 subjects (18 low-risk and 16 high risk subjects) that had no data points missing.

Regional effects obesity risk factors

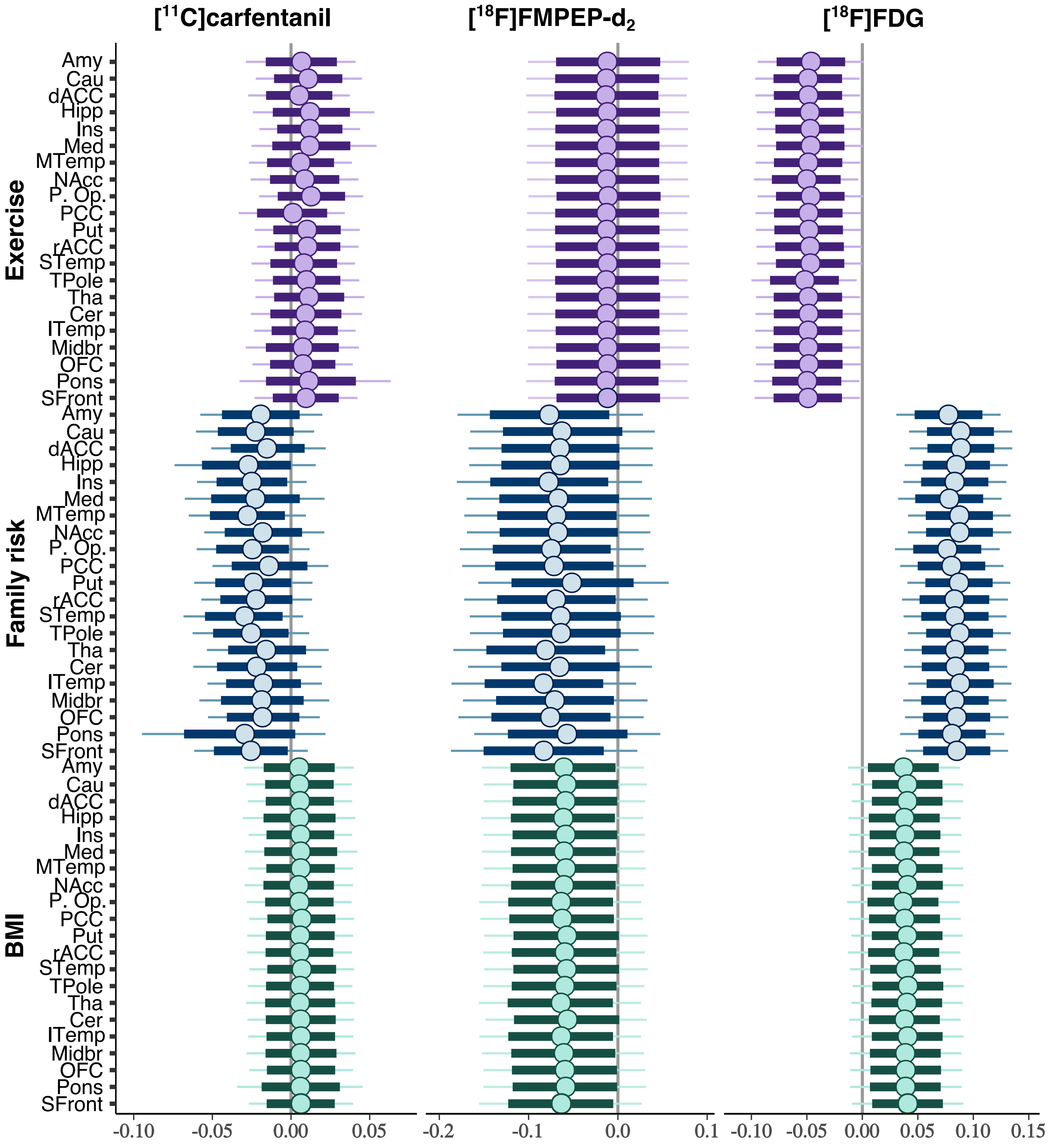

Supplementary Figure 2. Regional effects of obesity risk factors. Posterior distributions of the regression coefficients for exercise, family risk and body mass index (BMI) on log-transformed binding potential (BP_{ND}) of the [^{11}C]carfentanil, brain glucose uptake (BGU) quantified with [^{18}F]FDG and volume of distribution (V_T) of the [^{18}F]FMPEP-d_2 in 21 regions of interest, age as a covariate. The colored circles represent posterior means, the thick horizontal bars 80% posterior

intervals, and the thin horizontal bars 95% posterior intervals. Abbreviations stand for Amy = amygdala, Cau = caudate, dACC = dorsal anterior cingulate cortex, Hipp = hippocampus, Ins = insula, Med = medulla, MTemp = middle temporal gyrus, NAcc = nucleus accumbens, P.Op = pars opercularis, PCC = posterior cingulate cortex, Put = putamen, rACC = rostral anterior cingulate cortex, STemp = superior temporal gyrus, TPole = temporal pole, Tha = thalamus, Cer = cerebellum, ITemp = inferior temporal gyrus, Midbr = midbrain, OFC = orbitofrontal cortex, Pons = pons, SFront = superior frontal gyrus.

Serum anandamide and CB_1 receptor availability

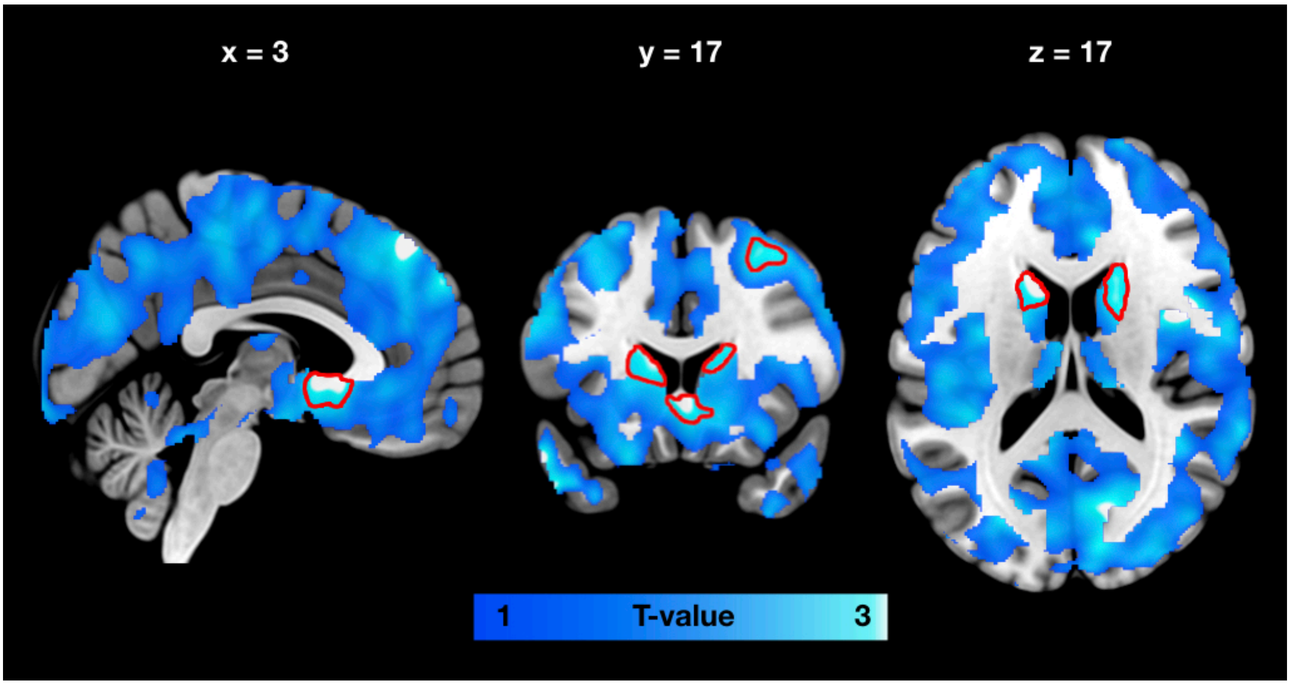

Supplementary Figure 3. Serum anandamide and CB_1 receptor availability. Brain areas where serum anandamide was associated with lowered brain CB_1 receptor availability in the 36 studied individuals (age corrected). The data are thresholded at $p < 0.05$, FWE corrected at cluster level. The areas marked with red denote clusters significant with Bonferroni-corrected cluster-defining p value (0.05/8 = 0.00625).

Linear regression analysis of family risk with three tracers

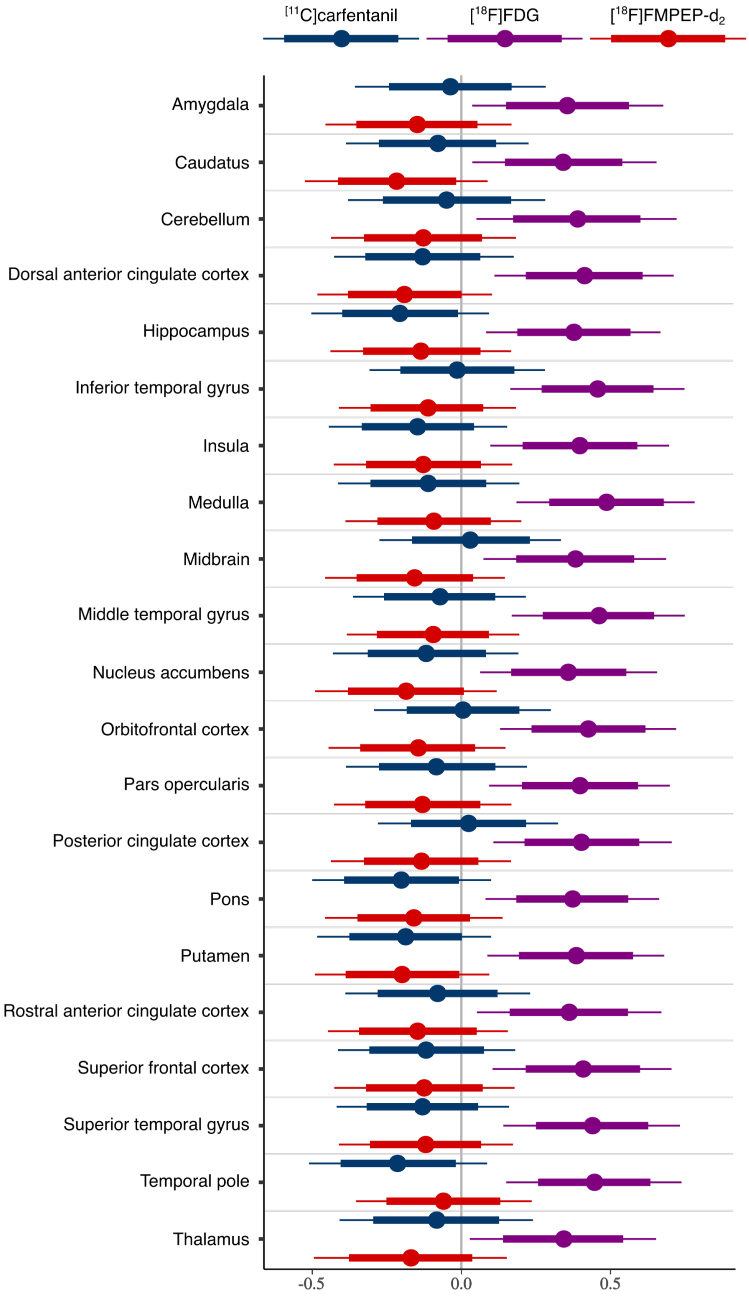

Supplementary Figure 4. Results of the Bayesian linear regression models of the familial obesity risk. [^{11}C]carfentanil binding potential (BP_{ND}), brain glucose uptake (BGU) in [^{18}F]FDG scans and [^{18}F]FMPEP-d_2 volume of distribution (V_T) as the predictors of familial obesity risk in 21 regions of interest. The model is calculated with the 34 subjects (18 low-risk and 16 high risk subjects) that had no data points missing. PET outcome measures were age-adjusted.

References

1. DeFronzo RA, Tobin JD, Andres R. Glucose clamp technique: a method for quantifying insulin secretion and resistance. *American Journal of Physiology-Endocrinology And Metabolism* 1979; **237**(3)**:** E214.

2. Innis RB, Cunningham VJ, Delforge J, Fujita M, Gjedde A, Gunn RN *et al.* Consensus nomenclature for in vivo imaging of reversibly binding radioligands. *Journal of cerebral blood flow and metabolism : official journal of the International Society of Cerebral Blood Flow and Metabolism* 2007; **27**(9)**:** 1533-9.

3. Lammertsma AA, Hume SP. Simplified reference tissue model for PET receptor studies. *Neuroimage* 1996; **4**(3)**:** 153-158.

4. Frost JJ, Douglass KH, Mayberg HS, Dannals RF, Links JM, Wilson AA *et al.* Multicompartmental Analysis of [11C]-Carfentanil Binding to Opiate Receptors in Humans Measured by Positron Emission Tomography. *Journal of Cerebral Blood Flow & Metabolism* 1989; **9**(3)**:** 398-409.

5. Logan J. Graphical analysis of PET data applied to reversible and irreversible tracers. *Nuclear Medicine and Biology* 2000; **27**(7)**:** 661-670.

6. Lahesmaa M, Eriksson O, Gnad T, Oikonen V, Bucci M, Hirvonen J *et al.* Cannabinoid type 1 receptors are upregulated during acute activation of brown adipose tissue. *Diabetes* 2018; **67**(7)**:** 1226-1236.

7. Thie JA. Clarification of a fractional uptake concept. *J Nucl Med* 1995; **36**(4)**:** 711-2.

8. Feng D, Huang S-C, Wang X. Models for computer simulation studies of input functions for tracer kinetic modeling with positron emission tomography. *International journal of bio-medical computing* 1993; **32**(2)**:** 95-110.

9. Wu H-M, Bergsneider M, Glenn TC, Yeh E, Hovda DA, Phelps ME *et al.* Measurement of the Global Lumped Constant for 2-Deoxy-2-[18F]Fluoro-D-Glucose in Normal Human Brain Using [15O]Water and 2-Deoxy-2-[18F]Fluoro-D-Glucose Positron Emission Tomography Imaging: A Method with Validation Based on Multiple Methodologies. *Molecular Imaging & Biology* 2003; **5**(1)**:** 32-41.

10. Snyder W, Cook M, Nasset E, Karhausen L, Howells GP, Tipton I. *Report of the task group on reference man*, vol. 23. Pergamon Oxford, 1975.

11. Dickens AM, Borgan F, Laurikainen H, Lamichhane S, Marques T, Rönkkö T *et al.* Links between central CB1-receptor availability and peripheral endocannabinoids in patients with first episode psychosis. *npj Schizophrenia* 2020; **6**(1)**:** 1-10.
